# High spectral specificity of local chemical components characterization with multichannel shift-excitation Raman spectroscopy

**DOI:** 10.1038/srep13952

**Published:** 2015-09-09

**Authors:** Kun Chen, Tao Wu, Haoyun Wei, Xuejian Wu, Yan Li

**Affiliations:** 1 Key Lab of Precision Measurement Technology & Instrument, Department of Precision Instrument, Tsinghua University, Beijing 100084, China; 2 Department of Physics, 366 Le Conte Hall MS 7300, University of California, Berkeley, California 94720, USA

## Abstract

Raman spectroscopy has emerged as a promising tool for its noninvasive and nondestructive characterization of local chemical structures. However, spectrally overlapping components prevent the specific identification of hyperfine molecular information of different substances, because of limitations in the spectral resolving power. The challenge is to find a way of preserving scattered photons and retrieving hidden/buried Raman signatures to take full advantage of its chemical specificity. Here, we demonstrate a multichannel acquisition framework based on shift-excitation and slit-modulation, followed by mathematical post-processing, which enables a significant improvement in the spectral specificity of Raman characterization. The present technique, termed shift-excitation blind super-resolution Raman spectroscopy (SEBSR), uses multiple degraded spectra to beat the dispersion-loss trade-off and facilitate high-resolution applications. It overcomes a fundamental problem that has previously plagued high-resolution Raman spectroscopy: fine spectral resolution requires large dispersion, which is accompanied by extreme optical loss. Applicability is demonstrated by the perfect recovery of fine structure of the C-Cl bending mode as well as the clear discrimination of different polymorphs of mannitol. Due to its enhanced discrimination capability, this method offers a feasible route at encouraging a broader range of applications in analytical chemistry, materials and biomedicine.

The spectral specificity of Raman spectroscopy has been recognized as a powerful tool for characterizing molecular structures for a wide range of applications, including physics, chemistry, biology and biomedicine^1^,^2^,^3^,^4^,^5^,^6^. Its spectral selectivity is mainly determined by the resolving power of the instruments. High resolution Raman spectroscopy can benefit its capability of charactering lifetime and phonons in acoustic cavities^4^, discriminating isotopic effects^5^ and even exploring single molecules via surface enhanced Raman scattering^6^. This spectral resolution issue has therefore attracted much attention and led to many practical techniques in recent years. Instrumental-method-based high resolution techniques normally involve the use of long focal distances^5^ or tandem configurations^7^ to achieve large dispersion and subsequently fine Raman structure. Unfortunately, there exists a severe trade-off between the achievable spectral resolution and the dispersion-loss of such schemes, because the amount of dispersion determines the spectral resolution. To improve the spectral specificity a signal can be dispersed only so much before it falls beneath the single-shot noise level. In turn, this drastically deteriorates the quality of the Raman spectra with signal-to-noise (SNR) decreases, and the results can be unstable.

Raman signatures become overlapped during acquisition as data pass through the dispersion and transmission processes because of the broadening effect of the instrumental response function. Because this degradation can be typically modeled by convolution with a blur kernel, deconvolution is recognized as being useful for spectroscopy to upgrade the spectral resolution^6^,^8^,^9^,^10^,^11^,^12^,^13^,^14^,^15^,^16^,^17^,^18^. The state-of-art methods can be classified into two major types: non-blind deconvolution (NBD) method^8^,^9^,^10^,^11^ and blind deconvolution (BD) method^12^,^13^,^14^,^15^,^16^,^17^,^18^. Current BD methods estimate the kernel and highly specific spectrum simultaneously from the measured data, without prior information. One major challenge is that all of the existing BD methods of spectral analysis are based on the single-framework, which is a well-known strongly ill-posed problem and vulnerable to noise. This drawback greatly limits its practical application. Apart from the broadening effect of the blur kernel, discretization of the detector may also degrade the Raman spectroscopic selectivity because in many optical systems the detector pixels are believed to be relatively large compared to the optical spot^19^. This issue is beyond the capacity of the deconvolution technique. In the 2-D case of image processing, a so-called super-resolution technique (SR), which has been established to retrieve the missed information caused by detector decimation, is one of the most promising and active research areas^19^,^20^,^21^,^22^. Drawing on the same concept, we can utilize a specific process of combining a sequence of low-resolution Raman spectra to produce a spectrum with higher spectral specificity. In this process, low-resolution spectra are subsampled as well as shifted with subpixel precision and the blurring function is assumed to be known or ignorable. The key point is that the subpixel shift is necessary to implement the SR technique to provide new information, which is the primary impediments. That is because the current subpixel shift always comes from the mechanical motion of the focal plane or detector and the precision is subject to mechanical tolerances^22^. This inaccuracy in turn can further deteriorate the recovery of the true signal, leaving a small misalignment behind.

This enhanced capability of deconvolution and the SR techniques are closely related theoretically. The goal of spectral deconvolution is to recover a degraded (e.g., blurred or noisy) spectrum, but it does not change the size of the spectrum. Although SR reconstruction can be considered a second-generation problem of spectra restoration, here we will still adopt the same term “super resolution” to refer to this subpixel processing approach toward resolution enhancement, like in the 2-D case. We attempted to find solutions to these problems that both techniques encounter. First, this strongly ill-posed property of current deconvolution methods should be addressed as a typical underdetermined inverse problem, where we have more unknowns (spectrum and blur) than equations, and it is highly sensitive to noise and minor perturbations and prone to numerical inaccuracy for large spectral data (see Supplementary Note 1.1). The ill-posed nature can be remedied to a great extent by simultaneously considering multiple spectra from multiple Raman acquisitions. Single-channel detection should be extended to form a multichannel framework and this idea has achieved great success in the 2-D case of imaging processing^23^,^24^,^25^. In this case, the problem is referred to as multichannel blind deconvolution (MBD). The MBD problem assumes that we have single input channel but *K* (*K* > 1) output channels. The outputs of each channel pair are linearly related by their channel responses. The use of such a cross relation between each output pair is the basic idea behind our deterministic blind multichannel identification where we can precisely recover the overlapping chemical specificity of Raman scattering. Missing Raman signatures in one channel can be supplemented by those in the other channels. This structure is not available in single-channel system (for details see Supplementary Note 1.2). The successful application of MBD depends on the difference between one channel and the other. From this perspective, we can modulate the entrance slit to form different detecting channel. More specifically, four slits of different shapes, including a single slit, double slit, cross-shaped slit and triangular slit, are applied in our research, elaborated further in the methods section.

Secondly, for the SR technique, only the low-resolution spectra have different subpixel shifts from each other, so the different chemical specificity contained in each low-resolution spectrum can be exploited to obtain a high specificity spectrum. The quality of the high resolution reconstruction is critically dependent on the accuracy of the registration of the spectrally (spatially) shifted, undersampled spectrum to a common reference frame. Here we establish a feasible and simple approach to achieving precise subpixel shifts in the acquisition process. Because a Raman shift in the photon frequency just depends on only the excitation or deactivation of molecular vibrations^26^, Raman peaks would track with the wavelength of the excitation laser, which means that the Raman spectrum is frequency-shifted by the amount of the excitation shift. This property has been widely used in shifted excitation Raman difference spectroscopy to subtract the fluorescence background^27^. Due to the linear transformation property, it contains a mathematical proof that shift-excitation exploited in our scheme is identical to the traditional subpixel shift method where both provide extra chemical specificity for reconstruction (for details, Supplementary Note 1.3).

Incorporating the slit-modulation and shift-excitation into a multichannel blind deconvolution and super-resolution scheme, we propose a method with high spectral specificity as well as enhanced discrimination capability, which is termed SEBSR. One can find a more detailed mathematical description in Supplementary Note 1.4. SEBSR accurately elaborates a single input multiple output (SIMO) formation model based on the multiple low-resolution acquisition processes, as summarized in Fig. 1(a). System degradation and detector decimation are both considered in this model, and this unifying approach presented here is the first attempt, to our knowledge, to solve BD and SR simultaneously in the spectral recovery. Multiple Raman acquisitions based on slit-modulation and shift-excitation are easily implemented in our experiments, illustrated in Fig. 1(b), where an optical frequency comb (OFC) is utilized to calibrate the excitation shifts to achieve the precise recovery of high resolution spectra.

In this article, we demonstrate that SEBSR uses a multichannel acquisition framework to facilitate high-resolution applications and enhance discrimination capability of Raman spectroscopy. We accomplish this unifying method by formulating the reconstruction problem as a minimization of a regularized energy function, where the regularization is carried out in both the spectra and blur domains. The main contribution of this work is the combination of blind deconvolution and super resolution, which lies in the application of multiple acquisitions and accurate subpixel shifts based on shift-excitation. It circumvents the fundamental trade-off between dispersion-loss and enhanced resolution. Synthetically blurred data with different but challenging SNRs were utilized to demonstrate the potential of the proposed approach and its superior performance. Its enhanced discrimination capability clearly benefits the recovery of the fine structure of the C-Cl bending mode as well as the distinction of different polymorphs of mannitol in our experiments.

## Results

As a demonstration of our technique, we first show a scenario of an experiment working with synthetically blurred data to evaluate the quality of high resolution spectra for different SNRs, qualitatively and quantitatively. As a comparison, another recently proposed deconvolution method, SBD^15^, which involves blind deconvolution but considers only a parametric model of blur, is also applied in our experiments. The fundamental idea of this article is to resolve more fine structures of Raman spectra to enhance its spectral specificity. We utilize Lorentzian expression to model a sequence of challenging Raman signals, as seen in Fig. 2(a), where both doublet and triplet fine structures are generated. Convolving with different kernels of 6 channels and accurately shifting, we obtain degraded Raman spectra, which become less resolved as well as low specificity, in Fig. 2(c).

Here, different kernels (more than Gaussian distribution) in Fig. 2(b) are applied in our simulation to demonstrate the potential of the SEBSR method. We should note that even though the overall instrumental response function is believed to approach a Gaussian form in many studies^12^, it is just an approximation to the real kernel. Because of this so, we wanted to achieve a more objective estimation by avoiding human intervention and two more complex distributions (double-Gaussian function) and a non-Gaussian distribution (triangle function) were used to simulate the practically uncertain cases. Random noise is inevitable in the acquisition process and an example of noisy Raman spectra with different SNRs based on the degraded data of channel 1 is also shown in Fig. 2(d).

Figure 3(a,b) show the recovered high chemical specificity of the reconstructed Raman spectra after processing with respect to different SNRs. SNR ~100 is taken as a typical case with low noise, whereas SNR ~20 represents a challenging situation that we often encounter practically. Compared with the degraded spectrum, the figures clearly show that SEBSR can consistently upgrade the resolution of the Raman spectra, irrespective of the noise level. All of the hindered signals have been faithfully retrieved, which significantly enhances the discrimination capability for different substances. For example, the doublet fine structure at 1661 cm^−1^ and 1691 cm^−1^ is successfully resolved and the peak at approximately 2535 cm^−1^ is split into a triplet structure. Other weak Raman signals at 1601 cm^−1^ and 2926 cm^−1^ are also well resolved. All of these emerging fine Raman signatures can be regarded as representative of the chemical specificity of local components. SBD can also provide Raman spectra that are more resolved than the degraded ones. However, its performances varies with the SNR. Raman peaks could be retrieved to some extent with SNR ~100, whereas the recovery results deteriorate dramatically in Fig. 3(b). It leads to distortions and false peaks in the deconvolved spectrum, and most of the Raman peaks are submersed in intense noise, especially at 2926 cm^−1^ (with a larger vision). Even moderate level noise, has a very serious impact on this recovery method. Similar results can be found in the discussions in ref. 15 and in other published investigations^12^,^13^,^14^. Nevertheless, SEBSR can suppress intense noise by simultaneously considering multiple degraded spectra. This considerably alleviates the ill-posed nature of blind deconvolution associated with prior methods. Apart from the specific information on the chemical composition of the samples based on resolved spectroscopy, quantitative determination and interpretation have been well-established by use of the intensity of Raman scattering. Hence, we should retrieve not only a specific Raman signature but also its real intensity in the quantitative application of fine Raman spectroscopy. Larger images of multiple-structure Raman peaks further illustrate the comparison between the true Raman spectrum and the reconstructed Raman spectra to give visual assessments of performance of the two different methods. The recovered intensity of the Raman peaks of SEBSR nearly coincide with true ones, showing a remarkably good match, whereas even in a high SNR case (~100), all of the peak intensities of SBD significantly deviate from the true ones. Considering the important role of Raman intensity in quantitative analysis, SEBSR can provide a more accurate estimation, yet SBD may mislead the interpretation of local chemical concentrations. In order to fairly evaluate the performance of SBD, the degraded spectrum in channel 1 without downsampling serves as the input data for SBD. If we apply SBD to the same undersampled spectra as SEBSR, the results will be far inferior than those presented in Fig. 3. That is, SEBSR actually achieves better enhanced discrimination capability with little information thanks to the sub-pixel shifts and multichannel framework. In addition, SBD works for only channels 1, 2 and 3, where the blurring kernels originally have a Gaussian distribution. Estimated kernels of the two methods are also illustrated and discussed in Supplementary Note 2.1.

We demonstrate the utility of SEBSR to obtain satisfactory results using only 6 acquisition channels, as illustrated in Fig. 3 with a low SNR ~20. Given the small scattering cross-section in the Raman transition process, SNR is likely to further deteriorate to below 20. Then, the detection of weak signals presents a more significant challenge in spectroscopy, especially when averaging cannot be used. SEBSR has the potential to suppress extremely intense noise by extending the acquisition channel. We further demonstrate the performance of SEBSR with low SNR below 20, namely, SNR = 10 and SNR = 6 in Supplementary Note 2.2. Here, we illustrate an example with the challenging SNR ~3 in Fig. 4. The Raman signals from both the degraded spectrum and the deconvolution result of SBD in Fig. 4(a) are barely recognizable. However, SEBSR can obtain consistent High chemical specificity Raman spectra, irrespective of the noise. Weak Raman signatures are distorted by spiky features to some degree when six channels are used, as presented in Fig. 4(b) with yellow line. To emphasize the noise-rejection capabilities of this technique, we conducted SEBSR with 12 acquisition channels in Fig. 4(b) with blue line. Enhanced chemical specificity of the Raman signals was achieved with the help of the extra channels. This improvement can be ascribed to the robustness of MBD framework, in which missing information about the original signals in one channel can be supplemented from the other channels. SEBSR also presents an alternative for the practically recorded Raman spectra with extremely low SNR so that more acquisition channels can help with the recovery of hidden signals.

In addition to the visual comparison, quantitative performance assessments are evaluated on the basis of the merits^28^ of *RMSE*, *CC*, and *WCC* for each method, as shown in Table 1. A small *RMSE* corresponds to a good match, whereas larger values of *CC* and *WCC* denote a better reconstruction. A detailed definition of the three parameters can be found in the Supplementary Note 3. When the signal is totally free from noise or the SNR is equal to 200, SBD obtains satisfactory *RMSE*, *CC* and *WCC*. However, for SNR below 100, *RMSE* sharply increases with the decrease of SNR. Accordingly, *CC* and *WCC* drop significantly, especially when the SNR approaches to 20 or below. However, SEBSR achieves almost the same evaluations and consistently retrieved results, regardless of the noise distortions. These quantitative evaluations are consistent with visual assessment in Figs 3 and 4. SEBSR therefore takes full advantage of the chemical specificity of Raman scattering and enables to specific detection of substances with overlapping Raman bands.

To begin the validation of SEBSR, we demonstrated the spectral specificity of different isotopes of chlorine atoms in carbon tetrachloride (CCl_4_) where slight differences in the number of neutrons is generally difficult to discriminate using Raman spectroscopy with normal resolution. We took six degraded Raman spectra acquired from six channels with different slits and shift-excitations, as shown in Fig. 5(a). All of the degraded spectra are less resolved; yet the contours and positions of the Raman peaks differ slightly from each other due to the variation of slits and shift-excitation. In contrast, the originally rough Raman peak at approximately 459 cm^−1^ is split into a triplet after the processing of SEBSR in Fig. 5(b). The additional peaks mainly result from the isotopic effect of the chlorine atoms in CCl_4_. As illustrated in Fig. 5(b), the left Raman peak at 456.07 cm^−1^ is attributed to the C-^37^Cl bending mode and the right one at 462.42 cm^−1^ is assigned to the C-^35^Cl mode, which is consistent with other reported observations^29^. In addition to the high resolution, the high precision of the reconstructed spectrum residing in the calibration of OFC can further benefit isotopic research. Because the separations of the multiple components of the splitting line depend on the concentration of each isotope (^37^Cl and ^35^Cl), a highly precise Raman spectrum provides a more accurate estimation of the atomic weight as well as the structure of the molecule (e.g., bond length and angle). When the SBD method is implemented, the degraded spectrum of channel 6 serves as the single input signal. Unfortunately, the 459 cm^−1^ band is not clearly resolved compared with the SEBSR case. In addition, the rejection of the noise of the green line is not as good as the red line, shown in Fig. 5(b).

We further demonstrate the spectroscopic selectivity of SEBSR in the discrimination of polymorphs. As a polymorphic excipient, mannitol is commonly used in the pharmaceutical formulation of tablets or granulated powders for oral use and exists in three polymorphic forms: alpha (*α*), beta (*β*) and delta (*δ*)^30^. The detection of polymorphs has become essential in the pharmaceutical industry because different modifications can have markedly different biopharmaceutical properties^31^. Raman spectroscopy is currently a potential tool to ascertain the same chemical composition of the various solid forms as well as to verify the structural differences and hence confirm the presence of polymorphism, due to its nondestructive and noninvasive property^32^. However, this analysis is always based on high spectral resolution to distinguish some slight differences in specific Raman peaks. The enhanced discrimination capability of SEBSR can benefit this issue. Six degraded Raman spectra were also acquired in Fig. 6(a), and only one significant Raman peak is observed at 878.7 cm^−1^, which is similar to the results in ref. 30,31. In that case, one can barely detect all of the polymorphs, due to difficulties because of the low resolution. However, this peak splits into three peaks at 864.5 cm^−1^, 873.9 cm^−1^ and 880.8 cm^−1^ after the SEBSR processing in Fig. 6(b). This triplet structure represent the *δ*, *β* and *α* form, respectively, and all of the polymorphs can be perfectly resolved and were found to be in good agreement with other observation^31^. The triplet-structure peak at approximately 500 cm^−1^ has been better resolved compared with the measured one. Moreover, the spectrum measured in channel 6 is also processed by SBD and the result is presented in Fig. 6(b) by a green line. In contrast, SBD does not take full advantage of the chemical specificity of Raman scattering and fails to specifically discriminate between different polymorphs with overlapping Raman bands. Both the measured spectra and the retrieved ones seem noisier than those of CCl_4_ because the Raman scattering cross-section of mannitol is smaller than that of CCl_4_.

## Discussion

The last two experiments demonstrate that SEBSR retrieves high spectral specificity from degraded and often noisy spectra, which would otherwise not be possible with a normal deconvolution method in the originally recorded spectra. From the results discussed, we believe that SEBSR has the potential to provide a precise analysis of isotopic effects, or it could be applied for the practical discrimination of polymorphs in the pharmaceutical processing in order to avoid the costly repercussions through the untimely appearance of high spectral specificity Raman spectroscopy. In the previously simulated case, SBD is found to recover satisfactory results successfully when the noise is free or negligible. On the contrary, this visible improvement on spectral specificity cannot be achieved in the experimental case, even when the measured spectra have high SNRs. SBD is implemented by carrying out blind deconvolution but considers only a parametric model of the blur kernel, so its estimation of the blur is fixed to a Gaussian distribution. This is a rather reasonable assumption in the simulated cases where the real blur kernel is set as a Gaussian function in advance. However, this assumption may not work in practical situations, as detailed in Supplementary Note 2.3. Without prior knowledge, SEBSR obtains superior chemical specificity, which can be partly ascribed to the objective estimations of kernels. Another advantage of SEBSR resides in the multichannel framework, where the missing chemical specificity in one channel can be supplemented by that in the other channels and noise-rejection capability can be greatly enhanced. In addition, SEBSR takes into account the discretization effect of the detector and introduces the downsampling operator to further enhance spectral resolution, which further benefits its discrimination capability.

Generally, SEBSR enables the preservation of scattered photons to retrieve hidden/buried chemical specificity from spectrally overlapping components. This is essential for practical application because the Raman scattering cross-section is believed to be very small. Large dispersion-based high resolution Raman spectroscopy is always inexorably linked to the extreme optical loss of scattered photons in complicated tandem dispersion and transmission processes, which causes a drastic deterioration in the quality of the Raman spectra. However, SEBSR uses multiple degraded spectra to beat the dispersion-loss trade-off and facilitate high-resolution applications and overcomes this fundamental problem. Satisfactory SNRs of our recorded spectra lie in this improvement. For various applications in general and tissue diagnosis and solid chemical agents distinction applications in particular, it is often required to resolve some spectrally overlapping components of interesting bands of Raman spectra measured experimentally with intense noise. In such situations, it is always desirable to improve the spectral specificity of the Raman characterization with respect to different SNRs. As we have experimentally demonstrated, fine Raman signatures otherwise hindered by a low-resolution instrument with a challenging noise level could be obtained using SEBSR. We believe that its enhanced discrimination capability enables a wide applicability to specific types of real samples, such as protein folding secondary structures, crystallinity, polymorphism, or the subtle changes in hydrogen bonding and intrinsic stress/strain. The technique is simple and straightforward in its design and implementation, and can reveal more structure and component information from scattered Raman photons with its exquisite chemical specificity. Furthermore, highly precise Raman spectra calibrated by an OFC can be conducive to the quantitative characterization of molecular structures. We therefore anticipate that this technique may also benefit other researches, like in chemistry, materials and biomedicine.

## Methods

### SEBSR Experiment

**Figure 1:**
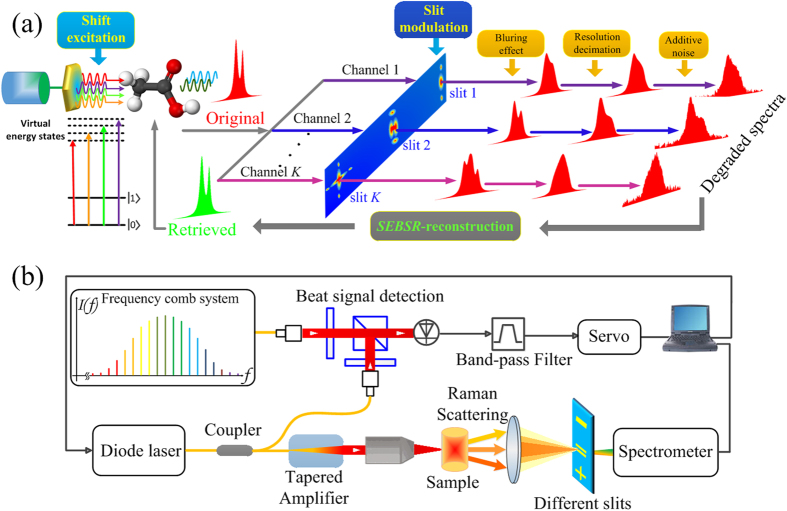
(**a**) Multichannel framework of acquisition and reconstruction. The original Raman spectrum is captured by *K* different channels which are subject to various degradations. It is modeled as outputs of *K* unknown channels driven by the single input. (**b**) Schematic representation of the SEBSR experimental set-up. Both of figures are drawn by Kun Chen.

**Figure 2:**
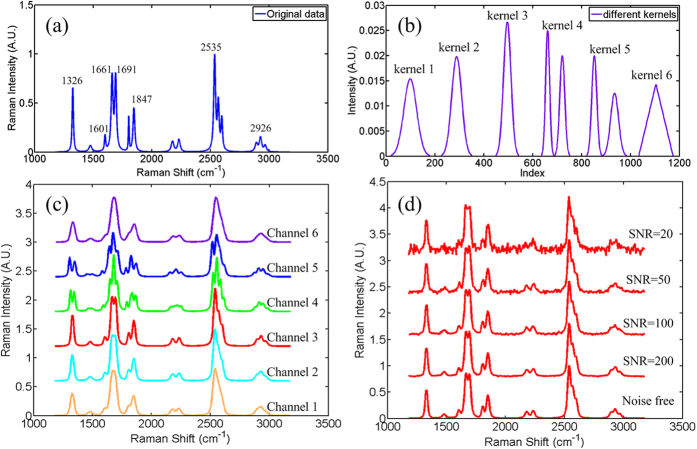
Mathematical example of simulated Raman spectra. (**a**) True (original) simulated Raman spectra (**b**) Six different simulated kernels. (**c**) Six degraded spectra acquired through six channels corresponding to different kernels in (**b**). (**d**) An example of noisy Raman spectra with different SNRs based on the degraded data of channel 1 in (**c**). For (**c**,**d**), all of the curves apart from the bottom one are vertically shifted for clarity.

**Figure 3:**
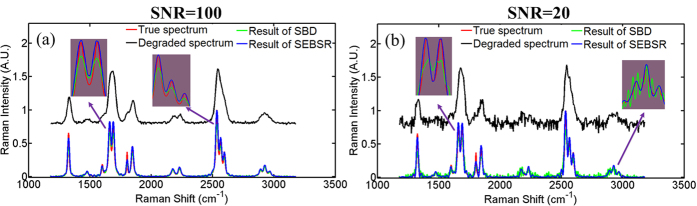
High chemical specificity of SEBSR and deconvolution results of SBD with respect to different SNRs. (**a**,**b**) correspond to SNR = 100 and SNR = 20, respectively. All of the black curves (degraded spectrum) are vertically shifted by 0.8 for clarity. Visual comparison of the true Raman spectrum and degraded ones with all of the retrieved results are also displayed.

**Figure 4:**
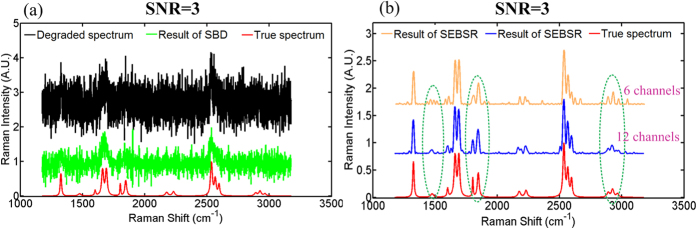
High chemical specificity of SEBSR with extremely low SNR ~3. (**a**) Deconvolution result of SBD. (**b**) Suppression of intense noise of SEBSR with different acquisition channels, 6 and 12. Visual comparison of the retrieved Raman signatures with the true ones in the dashed green circles presents better suppression of noise with more acquisition channels.

**Figure 5:**
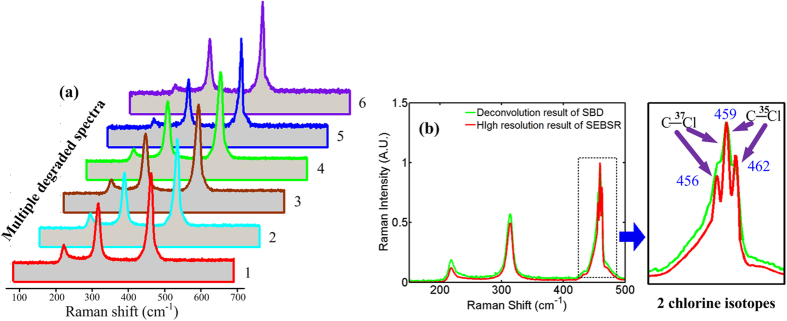
High spectral specificity of CCl_4_ Raman spectrum retrieved from experimental degraded data. (**a**) Six degraded spectra acquired with different slits and shift-excitations. (**b**) Reconstruction results of SEBSR (red line) and SBD (green line).

**Figure 6:**
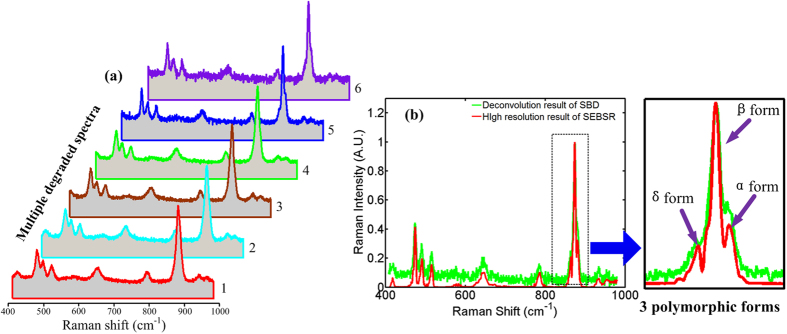
High spectral specificity of mannitol Raman spectrum retrieved from experimental degraded data. (**a**) Six degraded spectra acquired with different slits and shift-excitations. (**b**) Reconstruction results of SEBSR (red line) and SBD (green line).

To obtain shifted excitation wavelengths, an external cavity diode laser (ECDL, (Newport Corporation, TLB-6712, USA)) servers as the frequency-tunable excitation light source. However, the ECDL alone cannot provide high resolution because of the instability of its excitation wavelength and the inaccuracy of its shifted frequency intervals that directly transfers into the Raman spectral resolution. Thus, an OFC (Menlo System, FC1500-250, Germany), which is exploited as an optical frequency ruler, is employed to calibrate the frequency of the ECDL to improve the accuracy of the laser frequency-shifting interval. As shown in Fig. 1(b), the OFC consists of a series of discrete, equally spaced spectrum at the central wavelength of 780 nm generated by the second harmonic generation. Such a calibration is performed by measuring the beat frequency *f*_b_ between the frequency of the ECDL (*f*_ECDL_) and the nearest comb mode *N*, in which case the frequency of the spectroscopy laser is given by
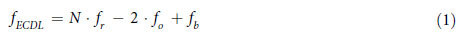
where the repetition rate (*f*_r_) and offset rate (*f*_o_) are locked to an Rb clock (Symmetricom, 8040C, USA) at 250 and 20 MHz, respectively. The comb mode integer *N* is ∼1,538,000 for the 780 nm excitation laser. The standard deviations of the repetition rate, offset rate, and beat frequency are 0.3 mHz, 0.5 Hz, and 0.6 Hz, respectively. Therefore, the combined standard uncertainty of the locked ECDL is 0.923 kHz. When converted to cm^−1^, it gives a high-accuracy frequency localization of better than 3.33 × 10^−8^ cm^−1^. More details of the ECDL locking system can be found in the ref. 33. More importantly, the line width of ECDL is less than 200 kHz, which is negligible compared with the repetition rate (*f*_r_ = 250 MHz). As a result, the excitation frequency *f*_ECDL_ can be easily and precisely shifted by changing the comb mode integer *N* in Eq. (1). The amount of the shifts can be determined by measuring the full width at half-maximum (FWHM) of the degraded Raman peak. For example, on-tenth FWHM or smaller is suitable to subpixel analysis. Note that the number of the shift-excitation *K* (also the number of output channels) and the downsampling factor *ε* should satisfy *ε*^2^ < *K* (for details, see Supplementary Note 1.3). In the practical recording process, each acquisition can be equipped with different silts and excitation shifts. After calibration by the OFC, the excitation laser is further injected into a Tapered Semiconductor Amplifiers (Newport Corporation, TA-6700, USA) to produce an amplified power of up to 500 mW. The Raman signals are analyzed with an imaging spectrograph (IsoPlane160, Princeton Instruments) and measured with a back-illuminated, deep depletion CCD (PIXIS1024BR, Princeton Instruments). As mentioned in Supplementary Note 1.2, successful application of MBD depends on the difference between one channel and the other. From this point of view, we conduct slit-modulation of the spectrograph to form different detecting channel. More specifically, four home-made slits with different shape, including single slit, double slit, cross-shaped slit and triangular slit, are applied in our research. What’s more, the width of single slit is also modulated and therein results in three levels: narrow (~15 *μ*m), moderate (*~*30 *μ*m) and broad (*~*50 *μ*m). It is worth noting that reducing the slit width will significantly deteriorates the quality of the spectra as well as the SNR. Therefore, we use only a relatively narrow slit (~15 *μ*m) to make a difference between the channels to show the potential of the proposed method. In the Supplementary Note 2.3, we discussed the real estimations of the systemic kernel from our experiments and have demonstrated that multiple spectra blurred in a slightly different way can be actually obtained. Liquid carbon tetrachloride and mannitol powder (XILONG Brand), made by Guangdong Xilong Chemical Reagents Company Limited, China, were also used in our study to investigate the performance of SEBSR.

### Mathematical solution to SEBSR

A high resolution Raman spectrum should be produced by combining a sequence of low-resolution spectra with the SIMO model below,
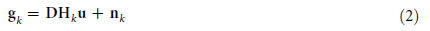


To solve the SEBSR problem, we adopt a classical approach of minimizing a regularized energy function (alternating minimizations, AM^34^). This way the method will be less vulnerable to noise and better posed. The energy consists of three terms and takes the form
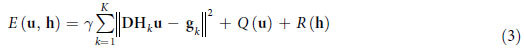


The first term measures the fidelity to the data and emanates from our acquisition model (3), where *γ* is the weight of the fidelity term. The remaining two are regularization terms with positive weighting constants that attract the minimum of *E* to an admissible set of solutions (for details, see Supplementary Note 1.4). It consists of two subproblems: minimization with respect to the spectra (*u*-step) and minimization with respect to the blurs (*h*-step). Both subproblems have some similarities because both the spectra and the blur regularization are not smooth and introduce nonlinearity into the problem. The algorithm first descends in the spectra subspace and after reaching the minimum, i.e., 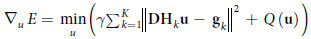 ((*u*-step), it advances in the blur subspace to achieve 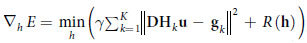 (*h*-step), and this scheme repeats. Specifically, we apply the Split-Bregman method^35^, which has achieved great success in L1-regularized problems, to the minimization of *u*-step and *h*-step. In conclusion, starting with some initial *h*_0_ the two iterative steps are:

### Require

input spectra (>2), blur size, parameters

1. : set *i* = 0, **h**^0^ is to delta functions and **u**^0^ is equal to the average of all of the input spectra
2. : Calculate *R*_Δ_
3. : repeat
4. : u^*i* + 1^ = u-step (u^*i*^, h^*i*^)
5. : h^*i* + 1^ = h-step (u^*i* + 1^, h^*i*^)
6. : *i* ← *i* + 1
7. : until stopping criterion is satisfied
8. : return u ← u^*i*^
9. The stopping criterion is 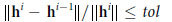.

## Additional Information

**How to cite this article**: Chen, K. *et al.* High spectral specificity of local chemical components characterization with multichannel shift-excitation Raman spectroscopy. *Sci. Rep.* **5**, 13952; doi: 10.1038/srep13952 (2015).

## Supplementary Material

Supplementary Information

## Figures and Tables

**Table 1:** Comparison of the performance of SEBSR and SBD with respect to different SNR.

**SNR**	**RMSE**		**CC**		**WCC**	
	**SEBSR**	**SBD**	**SEBSR**	**SBD**	**SEBSR**	**SBD**
Noise free	0.0212	0.0225	0.9955	0.9885	0.9928	0.9816
200	0.0216	0.0231	0.9954	0.9881	0.9927	0.9808
100	0.0260	0.0271	0.9921	0.9827	0.9908	0.9739
50	0.0275	0.0396	0.9921	0.9792	0.9890	0.9727
20	0.0289	**0.0563**	0.9856	**0.9459**	0.9876	**0.9388**
10	0.0343	**0.0802**	0.9728	**0.8618**	0.9704	**0.8978**
6	0.0364	**0.1228**	0.9680	**0.7445**	0.9652	**0.8350**
3	0.0380	**0.2251**	0.9663	**0.5085**	0.9636	**0.6898**
