# Supplementary material for: High spectral specificity of local chemical components characterization with multichannel shift-excitation Raman spectroscopy
Source: Sci Rep. 2015 Sep 9;5:13952. doi: 10.1038/srep13952 (PMC4563569; doi:10.1038/srep13952)
Supplement: Supplementary Information [file srep13952-s1.pdf]

SUPPLEMENTARY INFORMATION

High spectral specificity of local chemical components characterization with multichannel shift-excitation Raman spectroscopy

Kun Chen[1,3], Tao Wu[1], Haoyun Wei[1,], Xuejian Wu[2] & Yan Li[1]*

[1]Key Lab of Precision Measurement Technology & Instrument, Department of Precision Instrument, Tsinghua University, Beijing 100084, China

[2]Department of Physics, 366 Le Conte Hall MS 7300, University of California, Berkeley, California 94720, USA.

[3]chen-k13@mails.tsinghua.edu.cn

**Correspondence:*

Haoyun Wei, e-mail: luckiwei@mail.tsinghua.edu.cn

(Prepared for submission to *Scientific Reports*)

Supplementary Notes

Supplementary Notes 1. Theory

Note 1.1 ***General Mathematical model of deconvolution***
First, a proper mathematical model that describes the acquisition system is required. An original scene with a continuous intensity function can be firstly blurred by an unknown PSF and then geometrically shifted. Finally, the detector discretizes the spectra and further produces low-resolution and digitized noisy signals. In conclusion, the acquisition model becomes

$$g(v) = DT(h \otimes u)(v) + n(v) \qquad (1)$$

We formulate the problem in the discrete domain and use vector-matrix notation as follows,

$$\mathbf{g} = \mathbf{DTHu} + \mathbf{n} \qquad (2)$$

where g, h, u and n are the degraded spectrum, system PSF (kernel), original spectrum, and noise, respectively, and $\otimes$ denotes convolution. Matrices **H** performs convolution with h. **T** is a translation operator shifting a spectrum by t pixels. **D** is the downsampling operator that models the function of CCD sensors. It consists of convolution with the sensor PSF, followed by the sampling operator. Mathematically, the sensor PSF can be added to the system blur and **D** is generally regarded as the sampling operator. **D** can be further determined by a downsampling factor ε, which is a user-defined parameter to indicate the growth of the data size. Specifically, suppose that the data-length of degraded and reconstructed signals are p and q, respectively. So, $\varepsilon = q/p$.

The acquisition model in Eq. (2) embraces two distinct cases frequently encountered in literature. First, if the downsampling operator **D** and the geometric transform **T** are not considered, we face a blind deconvolution problem. Second, if the blur **H** is not considered or assumed known and **T** is suppressed up to a subpixel translation, we obtain a classical SR formulation. In practice, it is crucial to consider both two cases at once. Combining blind deconvolution and super-resolution, we are then confronted with a problem of shift-excitation blind super-resolution (SEBSR), which is the subject of this investigation. The approach presented in this chapter is the first attempts to solve SEBSR with only little prior knowledge in the spectral issue.

Note 1.2 ***MBD case***
The downsampling operator **D** and translation operator **T** are not present in Eq. (2) and it only convolves the input with blur kernel. Then we obtain a common degradation model,

$$g(v) = h(v) \otimes u(v) + n(v) \qquad (3)$$

or

$$\mathbf{g} = \mathbf{Hu} + \mathbf{n} \qquad (3)$$

This model accurately describes many common degradations and that justifies its frequent use[1-8]. Both the single-channel blind and non-blind deconvolution methods have been extensively studied and form a very successful branch. However, their applications are also limited since both algorithms are highly sensitive to noise and little perturbation and prone to numerical inaccuracy for large spectral data. In addition, blind deconvolution, as a typical inverse problem, is underdetermined as we have more unknowns (spectrum and blur) than equation and is always strongly ill-posed.

Extension of single-channel blind deconvolution approach forms a multichannel framework and this idea has achieved great success in 2-D case of imaging processing[9-11]. The ill-posed nature of blind deconvolution can be remedied to a great extent by considering multiple spectra. In this case, the problem is referred to as multichannel blind deconvolution (MBD) and will be the subject of our investigation. First, the MBD problem assumes that we have single input channel but K output channels and we can reformulate the acquisition model (3) as follows,

$$g_k = h_k \otimes u + n_k \quad 1 \le k \le K \qquad (4)$$

or

$$\mathbf{g}_k = \mathbf{H}_k \mathbf{u} + \mathbf{n}_k \qquad (4)$$

For any pair of two noise-free outputs g_i and g_j, given commutative property of convolution we can see that any two correct blurs h_i and h_j satisfy[12]

$$g_i \otimes h_j - g_j \otimes h_i = 0 \quad 1 \le i, j \le K \qquad (5)$$

The above equation shows that the outputs of each channel pair are linearly related by their channel responses. Clearly, if we have adequate data samples of the outputs, by (5), we can write out an overdetermined set of linear equations involving h_i and h_j. Under certain conditions which we will elaborate later, h_i and h_j can be determined uniquely up to a scalar multiple. The use of such a cross relation between each output pair is the basic idea behind our deterministic blind

multichannel identification. In other words, missing chemical specificity in one channel can be supplemented by that in the other channels. Note that this structure is not available in single-channel system.

More specifically, Eq. (5) can be rewritten as vector–matrix notation, using the commutative property of convolution,

$$\left[\mathbf{G}_i, -\mathbf{G}_j\right]\mathbf{h} = 0 \tag{6}$$

where $\mathbf{h} = [\mathbf{h}_i^{\mathrm{T}}, \mathbf{h}_j^{\mathrm{T}}]^{\mathrm{T}}$. Matrices $\mathbf{G}_i$ and $\mathbf{G}_j$ denote "valid" convolution with g_i and g_j, respectively. Considering all possible pairs of blurs, we can arrange the above relation into one system[10]

$$\mathcal{N}\mathbf{h} = 0 \tag{7}$$

where $\mathbf{h} = [\mathbf{h}_1^{\mathrm{T}}, \cdots, \mathbf{h}_K^{\mathrm{T}}]^{\mathrm{T}}$ and $\mathcal{N}$ is constructed solely by matrices that perform convolution with $g_1, \cdots, g_K$. From Eq. (7) we can see that the nullity (null-space dimension) is exactly 1 if the blur is correctly estimated. So, even the correct blur size is not known in advance in most real situations, we can precisely recover the blurs except to a scalar factor by applying SVD (singular value decomposition). When the channels are corrupted by noise, h can be estimated by solving a least squares problem shown below:

$$\min_h \left\|\mathcal{N}\mathbf{h}\right\|^2 \tag{8}$$

Blur and spectrum regularizations are also added to Eq. (8) to improve its stability. After that, Eq. (8) can be further reformulated as minimization of an energy function which is presented following.

It is worth noting that successful application of MBD depends on the difference between one channel and the other, which is defined as coprimeness in the 2-D case[11]. The condition of weakly coprime kernels may seem to be more problematic in 1-D case, where any kernel is decomposable. It is therefore likely that there might exist a factor common to all kernels. However, there are many situations where multiple spectra blurred in a slightly different way can be obtained. In fact, many studies have shown that the overall instrumental response function consists of multiple broadening factors, such as slit function, grating response, circuit response, etc.[4]. In principle, all rays with a given wavelength image the entrance slit plane onto exactly a specific point in the exit plane, thus transforming spectral information into spatial information. So, the configuration, shape and width, of the entrance slit affect the systematic PSF and spectral resolution significantly. From this point of view, we can modulate the entrance slit to form different detecting channel. More specifically, four slits with different shape, including single slit, double slit, cross-shaped slit and triangular slit are applied in our research. What's more, the width of single slit is also modulated and therein results in three levels: narrow, moderate and broad. It is worth noting that reducing the slit width will deteriorates the quality of spectra as well as SNR significantly. So, we just use a relatively narrow slit to make a difference between channels to show the potential of the proposed method.

Note 1.3 *SR case*

Super-resolution (SR) is a process of combining a sequence of low-resolution spectra in order to produce a higher resolution sequence. It is unrealistic to assume that the super-resolved spectrum can recover the original signals exactly. A reasonable goal of SR is a discrete version that has a higher spectral resolution than the measured ones and is free of the kernels (deconvolved). One key point of all the super-resolution techniques is that the quality of the reconstruction is critically dependent on the accuracy of the registration of the spectrally (spatially) shifted, undersampled spectra to a common reference frame. However, these shifts, originating from mechanical motion of the focal plane or detector, are always not known exactly due to mechanical tolerances and other equipment.

Here we establish a new approach to achieve precise sub-shifts in the SR process. The key point of the presented proposal should be addressed to the shift response of the Raman Effect to the slight excitation wavelength shifts. Since the Raman shift in photon frequency just depends upon excitation or deactivation of molecular vibrations[13], Raman peaks would track with the wavelength of the excitation laser, which means that the Raman spectrum is frequency shifted by the amount of excitation shift. This property has been widely used in shifted excitation Raman difference spectroscopy to subtract fluorescence background[14]. We denote shifted spectrum by $u_{shift}(v) = u(v - \Delta v)$ for Δv is the shifted of excitation in frequency. Now, we assume a known kernel $\hat{h}$ in Eq. (1),

$$\begin{aligned} g_{shift}(v) &= D(h \otimes u_{shift})(v) + n(v) = D(h(v) \otimes u(v - \Delta v)) + n(v) \\ &= D(h(v) \otimes \delta(v - \Delta v) \otimes u(v)) + n(v) \end{aligned} \tag{9}$$

In the vector-matrix notation, Eq. (9) becomes,

$$\mathbf{g}_{shift} = \mathbf{DHTu} + \mathbf{n} \tag{10}$$

Matrices $\mathbf{H}$ performs convolution with $\hat{h}$. $\mathbf{T}$ is a translation operator to shift the original Raman signals in the same manner as the delta function in Eq. (9). It is worth noting that shift-excitation is a typical linear transformation and $\mathbf{T}$ is invertible.

Then, $\mathbf{HT} = \mathbf{TT}^{-1}\mathbf{HT} = \mathbf{TH}$, where $\mathbf{H} = \mathbf{T}^{-1}\mathbf{HT}$. Given that $\mathbf{H}$ is a standard convolution with PSF $\hat{h}$ and $\mathbf{T}$ is linear, $\mathbf{H}$ remains a standard convolution but with $\hat{h}$ warped according to $\mathbf{T}$. Then we reformulate Eq. (10) as,

$$\mathbf{g}_{shift} = \mathbf{DTHu} + \mathbf{n} \tag{11}$$

The form of Eq. (11) accords with that of Eq. (2) and it also describes a typical SR model where the PSF is assumed to be known and $\mathbf{T}$ is suppressed up to a subpixel shift.

We have to keep in mind that if the low resolution spectra have different subpixel shifts from each other, the new chemical specificity contained in each low resolution spectra can be exploited to obtain a high spectral specificity spectrum. Here, we utilize a naive scheme in which the translation operator is determined by shift-excitation instead of mechanical motion, which guarantees the accuracy of spectral registration of the input channel, and the also the accuracy of reconstructed Raman signals to a common frame. Specifically, an external cavity diode laser (ECDL) calibrated by an optical frequency comb servers as the frequency-tunable excitation light source. The amount of shifts can be determined by measuring the full width at half-maximum (FWHM) of the degraded Raman peak. For example, on-tenth FWHM or smaller is suitable. Suppose that we have K output channels (K is also the number of shift-excitation). The downsampling factor ε which determines operator $\mathbf{D}$ should satisfy $\varepsilon^2<K$ and mathematical proof can be found in Ref. 15.

Note 1.4 ***SEBSR case***

So far, we have discussed the MBD and SR cases, respectively. In order to simultaneously estimate the blurs and highly specific spectrum, one can extend the SR problem to perform a naive approach by applying multichannel frame and formulating the problem as SEBSR. Introducing K output channels into Eq. (11), the acquisition model becomes:

$$\mathbf{g}_k = \mathbf{DT}_k\mathbf{H}_k^0\mathbf{u} + \mathbf{n}_k \tag{12}$$

where $\mathbf{T}_k$ is generated in k-th channel in the same manner as $\mathbf{T}$ in Eq. (11), and $\mathbf{H}_k^0$ is the unknown kernel of k-th channel. This model, like Eq. (4), elaborates a single input multiple output (SIMO) formation model. Even though the condition in Eq. (5) is not satisfied for the SEBSR case as the presence of downsampling operators ($\mathbf{D}$) violates the commutative property of convolution, the reconstruction of the PSF is possible in the SEBSR case, thanks to poly-phase formulation proposed by Filip Sroubek et al[10]. Correct PSFs are also available only on the basis of degraded and low-resolution spectra, like the case in Eq. (7). Theoretical verification can be further referred to Ref. 15. Here we will address our fundamental observation to the specific solution of Eq. (12).

As discussed in the SR case, the translation operator $\mathbf{T}_k$ is accurately determined by shift-excitation and suppressed up to a subpixel shift. Therefore, we denote a new version of the system blur $\mathbf{H}_k = \mathbf{T}_k\mathbf{H}_k^0$, where $\mathbf{H}_k$ performs convolution with the shifted version of the blur $\mathbf{H}_k^0$, and the acquisition model becomes

$$\mathbf{g}_k = \mathbf{DH}_k\mathbf{u} + \mathbf{n}_k \tag{13}$$

As a consequence of Eq. (10) and (13), we have that blurring a spectrally shifted spectrum is equivalent to blurring the original spectrum with a spectrally shifted point spread function. This result is important as it allows us to reformulate the problem as a SIMO frame even each input signals shifts a different amount. After that, we can readily extend the approach of MBD to SEBSR. Indeed, this should not come as a surprise since MBD and SR are related problems in our formulation.

Note 1.5 ***Mathematical processing of SEBSR***

In order to solve the SEBSR problem, we adopt a classical approach of minimizing a regularized energy function. This way the method will be less vulnerable to noise and better posed. The energy consists of three terms and takes the form

$$E(\mathbf{u},\mathbf{h}) = \gamma\sum_{k=1}^{K}\left\|\mathbf{DH}_k\mathbf{u} - \mathbf{g}_k\right\|^2 + Q(\mathbf{u}) + R(\mathbf{h}) \tag{14}$$

The first term measures the fidelity to the data and emanates from our acquisition model (13) and γ is the weight of the fidelity term. The remaining two are regularization terms with positive weighting constants that attract the minimum of E to an admissible set of solutions.

1. Regularization term for u

As Raman spectra is always expected to be smooth, it is reasonable to assume that the derivate of the spectra is generally sparse. Therefore, we use the L-1 term of first or second order difference of the spectra as the regularization term,

$$Q_1(\mathbf{u}) = \alpha\sum\left|\mathbf{D}_u\mathbf{u}\right| \tag{15}$$

$\mathbf{D}_u$ is the matrix that performs differentiate with u, and it can also be considered as a convolution matrix constructed with kernel [-1, 1] or [-1, 2, -1].

Another prior information we are certain of is $u \geq 0$. The function is

$$Q_2(\mathbf{u}) = \begin{cases} 0, & u \geq 0 \\ \infty, & else \end{cases} \quad (16)$$

The form seems to be quaint but it cooperates well with the Split-Bregman method[16] we use.
Thus, regularization term for *u* can be obtained as:

$$Q(\mathbf{u}) = Q_1(\mathbf{u}) + Q_2(\mathbf{u}) \quad (17)$$

2. Regularization term for *h*
As mentioned in Eq. (6) and (7), it is natural to utilize matrix $\mathcal{N}$. In the noiseless case, we can simply purpose $R(\mathbf{h})$ as

$$R(\mathbf{h}) = \|\mathcal{N}\mathbf{h}\|^2 \quad (18)$$

Yet the tiny noise could make *h* far from the null space of $\mathcal{N}$ and eq. (5) becomes:

$$g_i \otimes h_j - g_j \otimes h_i = n_i \otimes h_j - n_j \otimes h_i \neq 0 \quad (19)$$

So the correct *h* is not in the null space of $\mathcal{N}$. To eliminate the corruption caused by the noise, we can use the sparse property of kernels and apply an extra differentiate operator (L= [1,-3, 3, 1]). It leads to $L \otimes (g_i \otimes h_j - g_j \otimes h_i) = n_i \otimes L \otimes h_j - n_j \otimes L \otimes h_i \approx 0$. Here L is the third-order differentiate operator. Let Δ denote a matrix that performs convolution with L. We define also a modification of Eq. (6) as $R_\Delta = [\Delta\mathbf{G}_i, -\Delta\mathbf{G}_j]^{\mathrm{T}}[\Delta\mathbf{G}_i, -\Delta\mathbf{G}_j]$[29].
The regularization term for *h* is

$$R_1(\mathbf{h}) = \beta \mathbf{h}^{\mathrm{T}} R_\Delta \mathbf{h} \quad (20)$$

Another constraint we used is $h \geq 0$ as

$$R_2(\mathbf{h}) = \begin{cases} 0, & h \geq 0 \\ \infty, & else \end{cases} \quad (21)$$

$$R(\mathbf{h}) = R_1(\mathbf{h}) + R_2(\mathbf{h}) \quad (22)$$

This ensures we can get a valid solution and eliminates more ambiguity caused by over-estimated size of **h** and the factor property 1-D kernels. When extended to super resolution case, in most places the down sample matrix could be applied to matching the length of calculated data and the acquired data. When constructing $\mathcal{N}$, we need the length of *g* matching the calculated data, which is the SR factor larger than its original size. There are methods to precisely construct such a $\mathcal{N}$, but here we just enlarge *g* by inserting the former adjacent values. α and β are two regularization terms with positive weighting constants and that attract the minimum of *E* to an admissible set of solutions.

To find a minimizer of the energy function, we perform alternating minimizations (AM)[17] of *E* over **u** and **h**. The advantage of this scheme lies in its simplicity. It consists of two subproblems: minimization with respect to the spectra (*u*-step) and the minimization with respect to the blurs (*h*-step). Both subproblems share some similarities because both the spectra and the blur regularization are not smooth and introduce nonlinearity in the problem. The algorithm first descends in the spectra subspace and after reaching the minimum, i.e., $\nabla_u E = \min_u(\gamma \sum_{k=1}^{K} \|\mathbf{D}\mathbf{H}_k\mathbf{u} - \mathbf{g}_k\|^2 + Q(\mathbf{u}))$ (*u*-step), it advances in the blur subspace to achieve $\nabla_h E = \min_h(\gamma \sum_{k=1}^{K} \|\mathbf{D}\mathbf{H}_k\mathbf{u} - \mathbf{g}_k\|^2 + R(\mathbf{h}))$ (*h*-step), and this scheme repeats. Specifically, we apply the Split-Bregman method[16], which has achieved great success in L1-regularized problems, to the minimization of *u*-step and *h*-step. In conclusion, starting with some initial h_0 the two iterative steps are:

Require: input spectra (>2), blur size, parameters
1: set i=0, $\mathbf{h}^0$ is to delta functions and $\mathbf{u}^0$ is equal to the average of all the input spectra
2: Calculate R_Δ
3: repeat
4: $\mathbf{u}^{i+1}$ = *u*-step ($\mathbf{u}^i$, $\mathbf{h}^i$)
5: $\mathbf{h}^{i+1}$ = *h*-step ($\mathbf{u}^{i+1}$, $\mathbf{h}^i$)
6: $i \leftarrow i+1$
7: until stopping criterion is satisfied
8: return $\mathbf{u} \leftarrow \mathbf{u}^i$

The stopping criterion is $\|\mathbf{h}^i - \mathbf{h}^{i-1}\| / \|\mathbf{h}^i\| \leq tol$.

Supplementary Notes 2. Estimated blurring kernels of SEBSR and SBD

Note 2.1 ***Estimated kernels in simulated case***

As described in the main text, SBD is a Semi-Blind deconvolution method[7], where they carry out blind deconvolution but consider only a parametric model of blur kernel. It has achieved impressive improvements on the spectral resolution. Specifically, SBD, working on single input spectrum utilizes prior knowledge and blur kernel holds a fixed distribution of Gaussian form but with different variance. In addition, the downsampling process of detector is not considered in its reconstruction process. So, in order to evaluate the performance of SBD fairly, the degraded spectrum in channel 1 (Figure 2 in the main text) without downsampling serves as the input data for SBD, since its real kernel is a standard Gaussian function. However, in many optical systems the detector pixels are believed to be relatively large compared to the optical spot, resulting in a system that is undersampled. Because of this, recorded spectra are further discretized, thus more low-resolution. A downsampling operator **D**, introduced in SEBSR is used to restore real high specificity spectra. So for SEBSR, the input data is generated by further shifted and downsampled based on the input data of SBD. In this simulation, six low resolution spectra are generated by a decimation factor of ten which means SEBSR just needs one-tenth the input data of SBD for each spectrum. In the main text, the reconstruction performances of the two methods are investigated based on quantitative evaluation and visual assessment. In this supplementary section, we further describe the blur kernels simultaneously estimated from SEBSR and SBD to get deep insights into the difference between two methods.

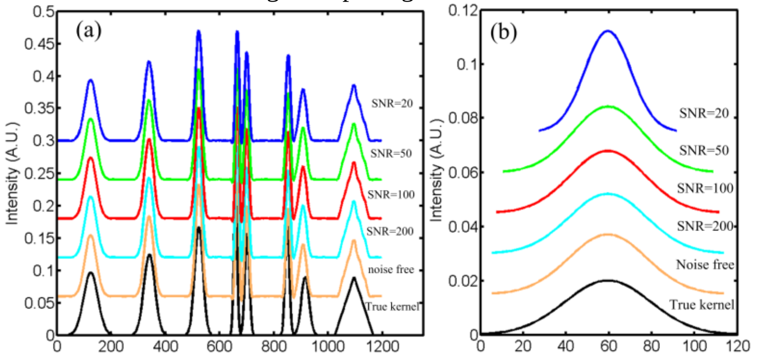

Figure S1. Estimated blur kernels of SEBSR and SBD. (a) Six estimated kernels of SEBSR with respect to different SNRs (b) Estimated standard Gauss kernel of SBD with respect to different SNRs. It corresponds to noise free, SNR=200, SNR=100, SNR=50 and SNR=20, respectively. All the curves apart from the bottom one are vertically shifted for clarity.

As shown in Fig. S1, it is clear that SEBSR attains a good match between the estimated kernels and the true ones, no matter how large the noise is. It is worth noting that SEBSR successfully get all the kernels back, not only the Gauss kernels but also the double-Gaussian and triangle distributions in channel 4, 5 and 6. It means that this method can handle with a more complex and practical situation. In contrast, kernel estimated by SBD varies with SNR and it more accords with the real one when the noise is low. That is intense noise generally causes significant deviation of the Gaussian size in turn can further deteriorate the recovery of true Raman signatures.

Note 2.2 ***Estimated kernels in simulated case***

We demonstrate the utility of SEBSR to obtain satisfactory results by using only 6 acquisition channels as illustrated in Fig.3 with low SNR ~ 20. Given the small scattering section in Raman transition process, SNR is likely to further deteriorate, thus below 20. Then the detection of weak signals presents a more significant challenge in spectroscopy, especially when averaging cannot be used. So, we further demonstrate the performance of SEBSR with low SNR below 20, that SNR=10 and SNR=6 in Fig. S2. The deconvolved results of SBD deteriorate so dramatically that one can hardly observe specific Raman peaks. However, SEBSR presents consistent retrieved Raman signals which are in good agreement with the true one. Quantitative assessments will be given below.

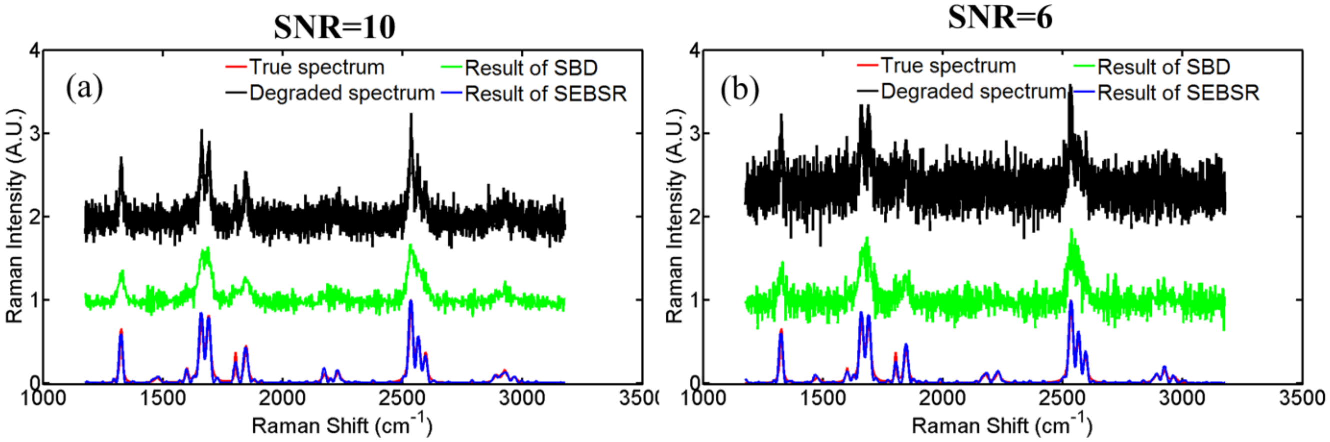

Fig. S2 High chemical specificity of SEBSR and deconvolution results of SBD with respect to different SNRs. (a) and (b) correspond to SNR=10 and SNR=6, respectively.

Note 2.3 ***Estimated kernels in experimental case***

Here, we further discuss the more objective and real estimations of systemic kernel from our experiments. It should be noted that the degraded spectrum of channel 6 severs as the single input signal for SBD that's because SBD does not consider any non-Gaussian kernel function and many studies have shown that the systemic kernel approaches to a Gaussian function with single normal slit. For CCl_4 case, all the six estimated kernels are shown in Fig. S3(a1) and vary from channel to channel. Narrow slits show smaller distribution area and double slits in channel 3 and 4 demonstrate asymmetric and multimodal distribution. These differences in turn demonstrate that multiple spectra blurred in a slightly different way can be actually obtained in our framework to satisfy the coprimeness of different channels. For mannitol, the six estimated kernels are also clearly different from each other. It is worth noting that the occurrence of the small spiky features on estimated kernels is ascribed to the relatively intense noise of the recorded spectra of mannitol. It is because of the fact that Raman scattering cross section of mannitol is smaller than that of CCl_4. Moreover, distinct difference of the estimated kernel between SEBSR and SBD can be found in both cases. Here, we want to address that the kernel estimated from SBD is a standard Gaussian function due to the prior assumption of a Gaussian parametric models of blur. However, the kernel estimations from SEBSR are hardly consistent with this assumption strictly, without prior knowledge. Obviously, the latter one is a more objective and reasonable by avoiding the direct human intervention to intrinsically specify the blur kernel.

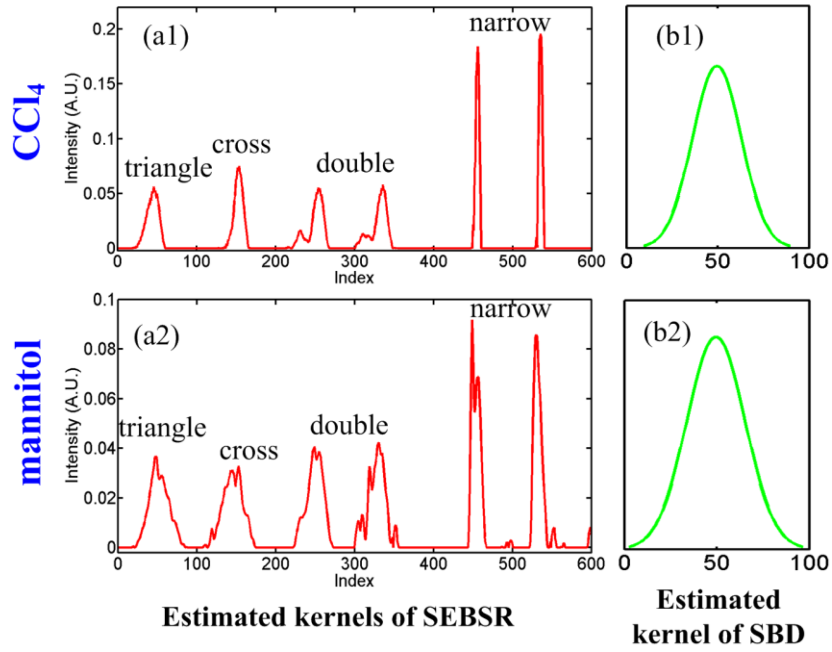

Figure S3. Estimated blur kernels of SEBSR and SBD. (a1) and (b1) are the results of CCl_4. (a2) and (b2) are the results of mannitol. (a1) and (a2) are the estimated kernels of SEBSR with different slits and excitation shifts: including triangular slit, cross-shaped slit, double slit and single narrow-width slit, respectively. (b1) and (b2) are the estimated kernels of SBD as a standard Gaussian distribution.

Supplementary Notes 3. Merits for quantitative performance Evaluation[18]

We denote R^o and R as the true spectrum and the recovered one. The root of mean square error (RMSE) is defined as

$$RMSE = \sqrt{\frac{\sum_{i=0}^{N}(R_i^o - R_i)^2}{N}} \qquad (23)$$

where N is the number of data points of the spectrum, and subscript i denotes the i-th data point.
The Pearson's correlation coefficient (CC) is

$$CC = \frac{\sum_{i=1}^{N}(R_i - \overline{R_i})(R_i^o - \overline{R_i^o})}{\sqrt{\sum_{i=1}^{N}(R_i - \overline{R_i})^2}\sqrt{\sum_{i=1}^{N}(R_i^o - \overline{R_i^o})^2}} \qquad (24)$$

And the self-weighted correlation coefficient [19] (WCC) is

$$WCC = \frac{\sum_{i=1}^{N} w_i (R_i - \overline{R_i})(R_i^o - \overline{R_i^o})}{\sqrt{\sum_{i=1}^{N} w_i (R_i - \overline{R_i})^2} \sqrt{\sum_{i=1}^{N} w_i (R_i^o - \overline{R_i^o})^2}} \quad (25)$$

where *w* is the weight array. $\overline{R_i}$ and $\overline{R_i^o}$ are the mean of corresponding spectra in *CC*, while they are weighted mean defined as $\overline{R_i} = \Sigma w_i R_i / \Sigma w_i$ and $\overline{R_i^o} = \Sigma w_i R_i^o / \Sigma w_i$ in *WCC*.

RMSE represents the average difference between the two sequences of signals, with a small *RMSE* corresponding to a good match. *Pearson's CC* represents the average similarity between the trends of the true and the recovered spectra, and the larger values denote a better match. As an improved version of *Pearson's CC, Griffiths' WCC* places emphasis on those Raman bands in the spectrum and thus can obtain a more reliable measure of the similarity, which is also consistent with visual comparison.

Supplementary Information References

1. Bowley, H. J. et al. The Fourier Self-Deconvolution of Raman spectra. *Appl. Spectrosc*. **39**, 1104-1009 (1985).
2. Jansson, P. A. Deconvolution: with applications in spectroscopy (Academic, 1984).
3. Lo ́renz-Fonfrı ́a, V. A. & Padro ́s, E. Maximum Entropy Deconvolution of Infrared Spectra: Use of a Novel Entropy Expression Without Sign Restriction. *Appl. Spectrosc*. **59**, 474-486 (2005).
4. Zou, M. Y. & Unbehauen, R. A deconvolution method for spectroscopy. *Meas. Sci. Technol*. **6**, 482-487 (1995).
5. Yuan, J., Hu, Z. & Sun, J. High-order cumulant-based blind deconvolution of Raman spectra. *Appl. Opt*. **44**, 7595-7601 (2005).
6. Yuan, J. & Hu, Z. High-Order Statistical Blind Deconvolution of Spectroscopic Data with a Gauss-Newton Algorithm. *Appl. Spectrosc*. **60**, 692-697 (2006).
7. Yan, L., Liu, H., Zhong, S. & Fang, H. Semi-blind spectral deconvolution with adaptive Tikhonov regularization. *Appl. Spectrosc*. **66**, 1334-1346 (2012).
8. Liu, H., Zhang, Z., Sun, J. & Liu, S. Blind spectral deconvolution algorithm for Raman spectrum with Poisson noise. *Photonics Research* **2**, 168-171 (2014).
9. Sroubek, F. & Flusser, J. Multichannel blind iterative image restoration. *Image Processing, IEEE Transactions on* **12**, 1094-1106 (2003).
10. Sroubek, F., Cristóbal, G. & Flusser, J. A unified approach to superresolution and multichannel blind deconvolution. *Image Processing, IEEE Transactions on* **16**, 2322-2332 (2007).
11. Sroubek, F. & Milanfar, P. Robust multichannel blind deconvolution via fast alternating minimization. *Image Processing, IEEE Transactions on* **21**, 1687-1700 (2012).
12. Liu, H., Xu, G. & Tong, L. A deterministic approach to blind identification of multi-channel FIR systems. Acoustics, Speech, and Signal Processing, 1994. ICASSP-94., 1994 IEEE International Conference on. IEEE. **4**, IV/581-IV/584 (1994).
13. D. A. Long. Raman Spectroscopy (McGraw-Hill, New York, 1977).
14. Cooper, J. B., Abdelkader, M. & Wise, K. L. Sequentially shifted excitation Raman spectroscopy: novel algorithm and instrumentation for fluorescence-free Raman spectroscopy in spectral space. *Appl. Spectrosc*. **67**, 973–984 (2013).
15. Sroubek, F., Flusser, J. & Cristobal, G. Multiframe blind deconvolution coupled with frame registration and resolution enhancement. In P. Campisi and K. Egiazarian (eds.), Blind Image Deconvolution: Theory and Applications. CRC Press, FL (2007).

16. Goldstein, T. & Osher, S. The split Bregman method for L1-regularized problems. *SIAM Journal on Imaging Sciences* **2**, 323-343 (2009).
17. Wang, Y., Yang, J., Yin, W. & Zhang, Y. A new alternating minimization algorithm for total variation image reconstruction. *SIAM Journal on Imaging Sciences* **1**, 248-272 (2008).
18. Chen, K., Wei, H., Zhang, H., Wu, T. & Yan, L. A Raman peak recognition method based automated fluorescence subtraction algorithm for retrieval of Raman spectra of highly fluorescent samples. A*nal. Methods 7*, 2770-2778 (2015).
19. Griffiths, P. R. & Shao, L. Self-Weighted Correlation Coefficients and Their Application to Measure Spectral Similarity. *Appl. Spectrosc*. **63**, 916-919 (2009).
